# Ductal stenting vs. surgical shunting in late presenting duct-dependent pulmonary circulation: a single-center experience

**DOI:** 10.3389/fcvm.2024.1382879

**Published:** 2024-04-19

**Authors:** Radityo Prakoso, Christine Nathalina Sinaga Simanjorang, Yovi Kurniawati, Brian Mendel, Budi Rahmat, Rita Zahara, Estu Rudiktyo, Damba Dwisepto Aulia Sakti, Renan Sukmawan

**Affiliations:** ^1^ Division of Pediatric Cardiology and Congenital Heart Disease, Department of Cardiology and Vascular Medicine, National Cardiovascular Centre Harapan Kita, Universitas Indonesia, Jakarta, Indonesia; ^2^ Department of Cardiology and Vascular Medicine, National Cardiovascular Centre Harapan Kita, Universitas Indonesia, Jakarta, Indonesia; ^3^ Department of Cardiology and Vascular Medicine, Sultan Sulaiman Government Hospital, Serdang Bedagai, Indonesia; ^4^ Division of Pediatric and Congenital Heart Surgery, Department of Surgery, National Cardiovascular Centre of Harapan Kita, Universitas Indonesia, Jakarta, Indonesia; ^5^ Division of Intensive and Cardiovascular Care, Department of Cardiology and Vascular Medicine, National Cardiovascular Centre Harapan Kita, Universitas Indonesia, Jakarta, Indonesia; ^6^ Division of Non-Invasive Diagnostic and Cardiovacular Imaging, Department of Cardiology and Vascular Medicine, National Cardiovascular Centre Harapan Kita, Universitas Indonesia, Jakarta, Indonesia

**Keywords:** ductal stenting, PDA stent, late presenter, mBTT shunt, palliative, pulmonary duct-dependent

## Abstract

**Introduction:**

PDA stenting is an option to mBTT shunt for younger patients; nevertheless, few reports of this palliative approach have been made for the late presenter population, especially for patients who are older than 30 days but under 5 years. This study aimed to evaluate the clinical result and intra-hospital costs of ductal stenting in late-presenting patients in comparison to surgical shunting.

**Methods:**

A single-center, retrospective cohort study was conducted from August 2016 to August 2022. This study included patients with pulmonary duct dependent CHD who were hospitalized for palliative therapy. The extracted data were baseline characteristics, clinical findings, supportive examination findings, complications, outcomes, and length of stay of the patients. Monitoring was carried out during treatment up to 30 days after the procedure.

**Results:**

A total of 143 patients were included in the analysis; 43 patients underwent PDA stent and 100 patients underwent mBTT shunt with median age of PDA stent group 110 (31–1,498) days and mBTT shunt group 174.5 (30–1,651) days. Primary outcome composite was not significant in both groups including 30 days mortality [6 (14%) vs. 14 (14%), *p* = 1.000], reintervention [1 (2.3%) vs. 7 (7%), *p* = 0.436], and 30 days rehospitalization [0 (0%) vs. 2 (2%), *p* = 0.319]. Secondary outcome analysis showed shorter ICU length of stay in the PDA stent group [2 (0–16) days vs. 4 (1–63) days, *p* = 0.002].

**Conclusions:**

PDA stent has an outcome that is non inferior from the mBTT shunt procedure in the composite outcome including 30 days mortality, reintervention, and 30 days rehospitalization but significantly lower in ICU length of stay.

## 1 Introduction

The initial palliative strategy of patients with duct-dependent pulmonary physiology remains challenging. During the last few decades, the m-BTT (modified Blalock-Thomas-Taussig) shunt was the first line of palliative treatment for this population, yet morbidity and mortality remain high (1, 2). Developments in the field of interventional pediatric cardiology have made it possible to do less invasive procedures, such as ductal stenting. This approach avoids the necessity of cardiopulmonary bypass (CPB), which is precarious during the newborn period, resulting in a shorter recovery phase. In a trial comparing the two palliative approaches, mortality in PDA stents was found to be non-inferior to m-BTT shunts, as were shorter hospital stays and less complications. However, the majority of these studies involve newborns (3–5).

In developing countries, there are very few cardiovascular centers with a team specialized to managing congenital heart surgery. This causes issues, including delays in the diagnosis of CHD patients, resulting in many newly diagnosed late presentations of CHD patient populations in the older age category, notably those above the age of one month; also, operational management necessitates a lengthy waiting period (6–9). Thus, this institutional investigation aims to explore the primary clinical endpoint, encompassing pre- and post-procedural saturations, intra-hospital mortality (both intra- and post-procedure), 30-day mortality, re-interventions, along with secondary clinical outcomes such as ICU, post-procedure, and overall hospital length of stay. It also scrutinizes intra-hospital and intra-procedure complications like acute thrombosis, bleeding, ductal spasm, stent dislodgement, postoperative infection, and pulmonary artery size. Moreover, the study assesses the intra-hospital costs associated with these two palliative approaches in late-presenting patients.

## 2 Methods

### 2.1 Study design and settings

A retrospective cohort study was conducted at a single center spanning from August 2016 to August 2022. The study received ethical approval from the Institutional Review Board of the National Cardiovascular Center Harapan Kita under the reference number LB.02.01/XX.2/8189/2022. The study included congenital heart disease patients with pulmonary duct dependency requiring palliative therapy, specifically PDA stent or m-BTT shunt without CPB, aged between 30 days and 5 years. Patients were categorized as either elective or emergency cases, with urgency determined by the need for the procedure within 24 h (cito) or 7 days (urgent). Exclusion criteria comprised patients with untreated infections before the procedure, those undergoing unifocalization of MAPCAs, and individuals without PDA in echocardiography diagnostic studies. Patient selection for PDA stent was personalized based on comorbidities, ductus arteriosus anatomy, and procedural risk. The electronic medical record (EMR) system maintained by the hospital documented all patients undergoing emergency or elective palliative therapy. Baseline demographic information, including sex, age, nutritional status, echocardiographic study data, and oxygen saturation, was extracted from the EMR. We employed the nutritional status classification developed by Wit et al. (10) specifically designed for low- and middle-income countries (10). All eligible research subjects will be followed from the initiation of the study until their discharge from the hospital.

### 2.2 Study outcome

The primary clinical endpoint encompasses pre- and post-procedural saturations, intra-hospital mortality, both intra- and post-procedure, 30-day mortality, and re-interventions such as stent re-dilation, re-stent for PDA stent procedures, and redo m-BTT shunt. Secondary clinical outcomes comprise ICU length of stay, post-procedure length of stay, overall hospital length of stay, as well as intra-hospital and intra-procedure complications, including acute thrombosis, bleeding, ductal spasm, stent dislodgment, postoperative infection, and pulmonary artery size. Z-scores for the right and left pulmonary arteries were derived from online nomograms utilizing Detroit Z-scores and were regarded as measures of the growth of each pulmonary artery individually (11). Intra-hospital complications defined as complications occurring during post-procedural care of PDA stent or MBTT shunt, including bleeding, stent/shunt failure due to thrombosis, spasm, or dislodgement, and infection. Intra-procedural complications defined as complications arising directly from palliative procedures, including bleeding, stent dislodgement, arrhythmia, and cardiac arrest. Dual antiplatelet therapy is administered in cases where two stents are involved in the procedure or when there are indications of high thrombogenicity, such as observed low flow during angiography, with the assessment being operator-dependent to determine the flow state. The dosage comprised 5 mg/kgBW of aspirin and/or 0.2 mg/kgBW of clopidogrel. Additionally, the study evaluates the intra-hospital costs associated with these two palliative strategies in late-presenting patients. At discharge, the patient was given ramipril 0.05 mg/kgBW, bisoprolol fumarate 0.2–0.4 mg/kgBW, and furosemide 1 mg/kgBW once daily if there were signs of congestion. The patient was then followed-up for six months.

### 2.3 Statistical analyses

The numerical data underwent the Kolmogorov–Smirnov normality test. Categorical data is presented as numbers (*n*) and percentages (%). Normally distributed numerical data is described using the mean value and standard deviation (mean ± SD), while non-normally distributed numerical data is presented as the median value along with the minimum and maximum values [median value (minimum—maximum)]. Categorical clinical outcomes will be analyzed using the *χ*^2^ test to assess differences in proportions between the two groups or Fisher's exact test if the requirements are not met. Numerical clinical outcomes will be subjected to an independent *t*-test for normally distributed data and the Mann–Whitney test for non-normally distributed data. Secondary outcomes showing significance in bivariate analysis underwent multivariate analysis using the linear regression test. Survival analysis was conducted using Cox regression and Kaplan–Meier methods. Statistical analysis utilized the Statistical Package for Social Science (SPSS) 26.0 software (12), with significance set at a *p*-value <0.05.

## 3 Results

### 3.1 Baseline characteristics

This study enrolled 143 patients who underwent PDA stent and m-BTT shunt procedures between August 2016 and August 2022. The final analysis included 43 patients with PDA stents and 100 patients with mBTT shunts. Baseline characteristics are summarized in Table 1, revealing significant differences in anatomic diagnosis, procedural status, and the use of antiplatelets during outpatient care. The primary diagnoses treated with palliative interventions were PAVSD in 21 patients (48.8%) in the PDA stent group and 71 patients (71%) in the mBTT shunt group, with the procedures often performed urgently. Dual antiplatelet therapy (DAPT) was more commonly used after PDA stent procedures compared to mBTT shunts.

**Table 1:** Baseline characteristics of 143 patients underwent PDA stents and m-BTT shunt procedures.

Variable	PDA *stent* (*n* = 43)	m-BTTs (*n* = 100)	*p*-value
Sex			
Male (*n*, %)	22 (51.2%)	56 (56.0%)	0.727
Female (*n*, %)	21 (48.8%)	44 (44.0%)	
Age at intervention, days (median, min-max)	110 (31–1,498)	174.5 (30–1,651)	0.171
Weight, kg (median, min-max)	4.6 (2.1–13.0)	5.5 (2.9–16.0)	0.067
Nutritional status			0.511
Normal (*n*, %)	16 (37.2%)	47 (47.0%)	
Low (*n*, %)	14 (32.6%)	30 (30.0%)	
Very low (*n*, %)	13 (30.2%)	23 (23.0%)	
Diagnosis			0.002
Ebstein anomaly (*n*, %)	2 (4.7%)	0 (0.0%)	
PA CAVSD (*n*, %)	2 (4.7%)	13 (13.0%)	
PA VSD (*n*, %)	21 (48.8%)	71 (71.0%)	
PA IVS (*n*, %)	16 (37.2%)	12 (12.0%)	
TA PA (*n*, %)	1 (2.3%)	1 (1.0%)	
TA PS severe (*n*, %)	1 (2.3%)	3 (3.0%)	
Procedural Status			<0.001
Cito (*n*, %)	14 (32.6%)	8 (8.0%)	
Urgent (*n*, %)	22 (51.2%)	52 (52.0%)	
Elective (*n*, %)	7 (16.3%)	40 (40.0%)	
*Stent*/*Shunt* diameter, mm (median, min-max)	4 (3–4)	4 (3–5)	0.126
Antiplatelet			
SAPT (*n*, %)	33 (76.7%)	98 (98.0%)	<0.001
DAPT (*n*, %)	6 (13.9%)	1 (1.0%)	0.003
Prostaglandin use before intervention (*n*, %)	8 (18.6%)	30 (30.0%)	0.227
Other intervention (*n*, %)	17 (39.5%)	22 (22.0%)	0.051
BAS (*n*, %)	14 (32.6%)	0 (0.0%)	
BPV (*n*, %)	2 (4.7%)	0 (0.0%)	
IAS stenting (*n*, %)	1 (2.3%)	0 (0.0%)	
PDA ligation (*n*, %)	0 (0.0%)	20 (20.0%)	
PDA Banding (*n*, %)	0 (0.0%)	1 (1.0%)	

BAS, balloon atrial septostomy; BPV, balloon pulmonary valvotomy; CAVSD, complete atrioventricular septal defect; DAPT, dual antiplatelet; IAS, interatrial septum; IVS, intact ventricular septum; m-BTTs, modified Blalock-Thomas-Taussig shunt; PA, pulmonary atresia; PDA, patent ductus arteriosus; PS, pulmonary stenosis; SAPT, single antiplatelet; TA, tricuspid atresia.

* *p < *0.05, significantly different between two groups.

### 3.2 Clinical outcomes

Pre-procedural oxygen saturation levels for PDA stent and mBTT shunt were 65% (range: 21%–86%) and 64% (range: 30%–86%), respectively, with no significant difference observed (*p* = 0.405). Post-procedural oxygen saturation levels were 92% (range: 40%–100%) and 82% (range: 50%–98%) for PDA stent and mBTT shunt, respectively, with a trend towards significance (*p* = 0.090). The primary composite outcome (death, reintervention, or rehospitalization within 30 days) occurred more frequently in the m-BTT shunt group (23% vs. 16.3%, *p* = 0.496, Table 2). Although mortality was 14% in both groups, the difference was not statistically significant (*p* = 1.000). Mortality causes in the PDA stent group included stent thrombosis and spasm in PDA (2), infection (2), and heart failure (2). In the m-BTT shunt group, mortality causes included life-threatening arrhythmia (2), sepsis (4), shunt thrombosis (1), cardiogenic shock (1), overshunted (2), acute respiratory distress syndrome/ARDS (1), and low cardiac output syndrome/LCOS (3). There were no cases of pulmonary artery discontinuity or LPA coarctation observed in either the PDA stent or mBTT shunt groups.

**Table 2:** Comparison of primary outcomes and secondary outcomes based on palliative strategy.

Variable	PDA *stent* (*n* = 43)	m-BTTs (*n* = 100)	*p*-value
Primary outcomes			
Pre-procedural oxygen saturation, % (median, min-max)	65 (21–86)	64 (30–86)	0.405
Post-procedural oxygen saturation (median, min-max)	92 (40–100)	82 (50–98)	0.090
Composite primary outcome (*n*, %)	7 (16.3%)	23 (23.0%)	0.496
Mortality within 30 days (*n*, %)	6 (13.9%)	14 (14.0%)	1.000
Unplanned reintervention (*n*, %)	1 (2.3%)	7 (7.0%)	0.436
Rehospitalization within 30 days (*n*, %)	0 (0.0%)	2 (2.0%)	0.319
Secondary outcomes			
ICU LOS duration, days (median, min-max)	2 (0–16)	4 (1–63)	0.002
LOS after procedure, days (median, min-max)	8 (0–38)	10 (0–112)	0.140
Total hospital LOS duration, days (median, min-max)	11 (5–43)	16.5 (2–128)	0.051
Other outcomes			
Complication			
Intraoperative complication (*n*, %)	1 (2.3%)	2 (2.0%)	1.000
Intrahospital complication (*n*, %)	3 (6.9%)	6 (6.0%)	1.000
Infection (*n*, %)	12 (27.9%)	30 (30.0%)	0.959

ICU, intensive care unit; LOS, length of stay; m-BTTs, modified Blalock-Thomas-Taussig shunt; PDA, patent ductus arteriosus.

* *p < *0.05, significantly different between two groups.

Regarding secondary outcomes, the ICU length of stay was significantly shorter in the PDA stents group (2 vs. 4 days, *p* = 0.002). The total hospital length of stay was shorter in the PDA stents group (11 vs. 16.5 days, *p* = 0.051), but this difference did not reach statistical significance. Infection complications occurred more frequently in the m-BTT shunt group, although the difference was not statistically significant (30% vs. 27.9%, *p* = 0.959). The PDA stent procedure demonstrated a shorter ICU length of stay than the m-BTT shunt [2.5 (0–16) vs. 4 (1–63), *p* = 0.001], with no differences in the length of stay after the procedure and total hospital length of stay.

**Figure 1:**
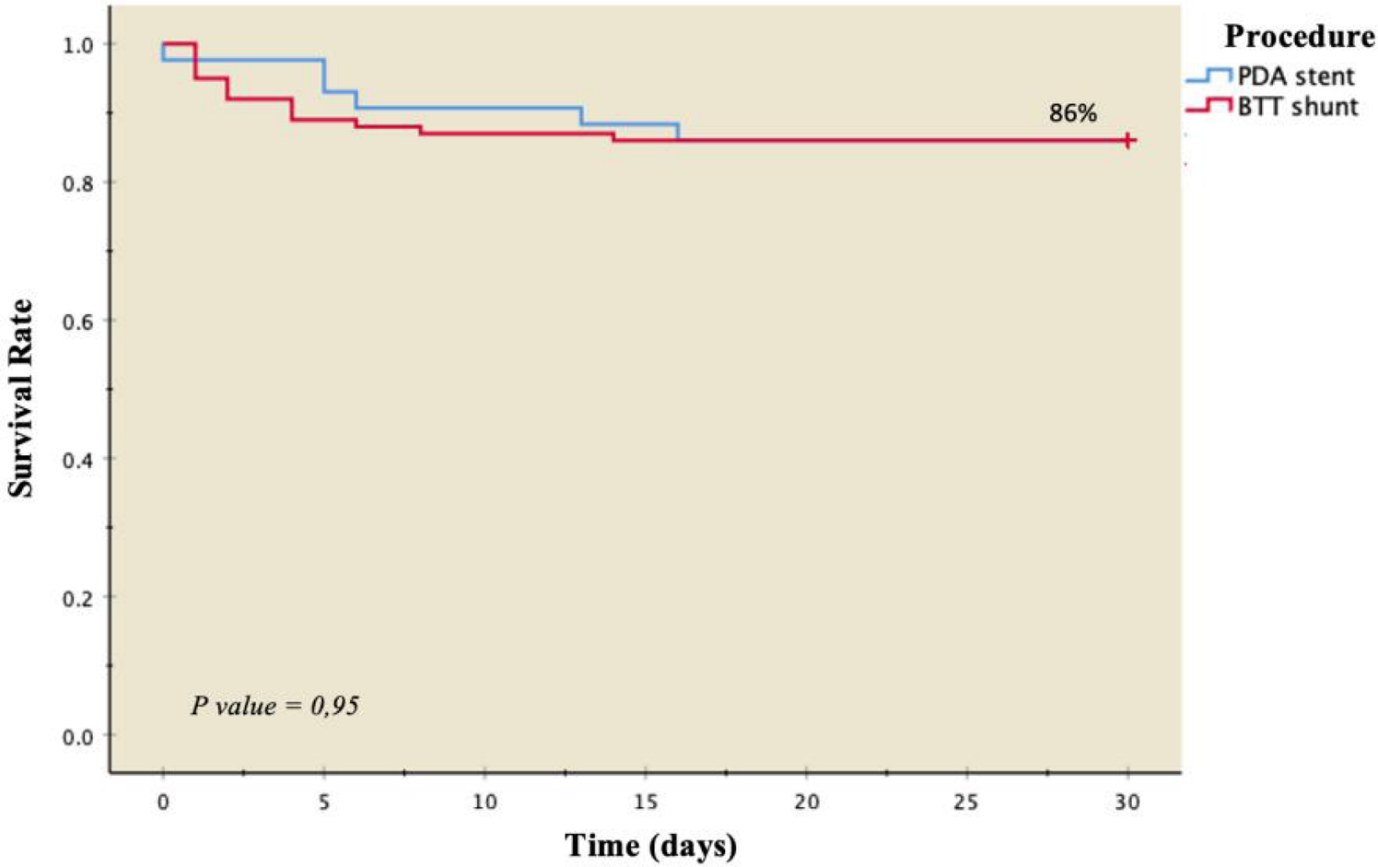
Kaplan–Meier survival curve based on treatment strategy.

Pulmonary artery measurements in 42 patients revealed both PDA stent and m-BTT shunt procedures providing favorable pulmonary artery growth. No differences were observed in the increase of pulmonary artery size between the two groups measured by McGoon's ratio (*p* = 0.10, Table 3). Kaplan–Meier survival curves based on primary outcomes showed a similar probability of survival between the two groups at 86%. PDA stents exhibited better survival rates from day 1 to day 16 post-operatively; however, after the 16th day, the survival probabilities were comparable (Figure 1).

**Table 3:** Comparison of pulmonary artery size PDA *stent* vs. mBTT *shunt.*

Variable	PDA *stent* (*n* = 13)	mBTT shunt (*n* = 29)	*p*-value
Pulmonary artery size			
RPA			
RPA-pre, mm (median, min-max)	5.0 (3.6–6.8)	5.0 (3.2–11.7)	
RPA-pre z-score (mean ± SD)	−0.62 ± 1.37	0.13 ± 2.43	0.239
RPA-post, mm (median, min-max)	9.4 (4.6–13.2)	8.7 (2.9–13.5)	
RPA-post z-score (mean ± SD)	2.48 ± 2.39	0.87 ± 2.89	0.187
% Increased (median, min-max)	67.4 (21.1–255.7)	25.3 (−31.1–127.0)	0.060
*p-*value (within group)	<0.001	<0.001	
LPA			
LPA-pre, mm (median, min-max)	4.2 (2.0–8.8)	4.5 (2.2–8.2)	
LPA-pre z-score (mean ± SD)	−1.28 ± 0.96	−1.16 ± 2.20	0.158
LPA-post, mm (median, min-max)	7.6 (3.0–15.3)	7.3 (3.0–10.9)	
LPA-post z-score (mean ± SD)	2.56 ± 2.49	1.09 ± 2.14	0.486
% Increased (median, min-max)	107.4 (3.0–220.2)	42.8 (−36.3–235.4)	0.110
*p-*value (within group)	0.001	<0.001	
McGoon ratio			
McGoon pre (median, min-max)	1.3 (0.9–2.4)	1.3 (0.7–2.0)	
McGoon post (median, min-max)	2.0 (1.3–2.9)	1.6 (0.9–2.6)	
% Increased (median, min-max)	42.2 (−24.9–147.4)	16.6 (−30.3–112.2)	0.100
*p-*value (within group)	0.008	0.001	

LPA, left pulmonary artery; m-BTTs, modified Blalock-Thomas-Taussig shunt; PDA, patent ductus arteriosus; RPA, right pulmonary artery.

* *p < *0.05, significantly different between two groups.

### 3.3 Overall intra-hospital cost

The median total intra-hospital cost in the PDA stent group was Rp. 84,296,407 (Rp. 59,285,973–Rp. 163,521,503), while the m-BTT shunt group had a median cost of Rp. 95,239,370 (Rp. 64,231,215-Rp. 565,143,693, *p* = 0.035). Differences in room costs [Rp. 14,300,000 (Rp. 3,500,000–Rp. 59,700,000) vs. Rp. 21,100,000 (Rp. 5,100,000–Rp. 296,000,000), *p* = 0.035], laboratory costs [Rp. 7,172,000 (Rp. 1,745,000–Rp. 22,085,000) vs. Rp. 11,062,500 (Rp. 3,290,000–Rp. 210,508,693), *p* = 0.006], and imaging costs [Rp. 480,000 (0–Rp. 2,000,000) vs. Rp. 800,000 (Rp. 320,000–Rp. 8,550,000), *p* < 0.001] were significant. No significant differences were found in service costs (see Table 4).

**Table 4:** Comparison of intra-hospital *cost of care* of PDA *stent* vs. mBTT *shunt.*

Variable	PDA *stent* (*n* = 43)	mBTT shunt (*n* = 100)	*p*-value
Intra-hospital *Cost of Care*			
Total costs (median, min-max)	Rp. 84,296,407 (Rp. 59,285,973–Rp. 163,521,503)	Rp. 95,239,370 (Rp. 64,231,215–Rp. 565,143,693)	0.039
Room costs (median, min-max)	Rp. 14,300,000 (Rp. 3,500,000–Rp. 59,700,000)	Rp. 21,100,000 (Rp. 5,100,000–Rp. 296,000,000)	0.004
Service costs (median, min-max)	Rp. 62,304,161 (Rp. 45,141,092–Rp. 109,312,503)	Rp. 59,052,998 (Rp. 46,519,215–Rp. 210,508,693)	0.912
Laboratory costs (median, min-max)	Rp. 7,172,000 (Rp. 1,745,000–Rp. 22,085,000)	Rp. 11,062,500 (Rp. 3,290,000–Rp. 50,085,000)	0.001
Imaging costs (median, min-max)	Rp. 480,000 (Rp. 0–Rp. 2,000,000	Rp. 800,000 (Rp. 320,000–Rp. 8,550,000)	<0.001

Currency is written in Indonesian Rupiah. Numerical data that is normally distributed is expressed in the form of mean ± standard deviation (SD), and data that is not normally distributed is expressed in the form of median (min-max). A variable is considered significant if the *p*-value is less than 0.05.

## 4 Discussions

### 4.1 Palliative strategies in late-presenter patients

Traditionally, the preferred method for palliation has been the surgical modified Blalock-Thomas-Taussig (mBTT) shunt, which, unfortunately, carries a notable morbidity and mortality rate (10%–20%) that remains unchanged over time. An appealing alternative has emerged in the form of transcatheter patent ductus arteriosus (PDA) stenting. The minimally invasive nature of PDA stenting eliminates the need for open-heart surgery, median sternotomy, and cardiopulmonary bypass (13–16). Notably, Ratnayaka et al. (15) shifted their practice from selective to universal PDA stenting for all patients requiring palliation with ductal-dependent blood flow. Contraindications such as PDA tortuosity, small pulmonary artery size, pulmonary artery discontinuity, being at risk for pulmonary artery discontinuity, or concerns about PDA stent bronchus compression are no longer considered.

In our investigation, several key findings emerged in late-presenter patients: (1) There were no significant differences in intra-hospital and 30-day post-operative mortality between the two palliative treatment groups, (2) PDA stents demonstrated a shorter ICU stay compared to m-BTT shunts, although the post-operative and total length of hospital stay did not exhibit significant differences between the two groups, (3) The occurrence of intra-hospital and intra-operative complications did not significantly differ between the two groups, and (4) Pulmonary artery growth associated with PDA stenting was comparable to that observed with m-BTT shunts. In one case, we document an increase from 5.04 to 9.4 (nearly doubled) before and after the insertion of a PDA-stent. We propose that in this case, both the hemodynamic impact of vasodilation and heightened blood flow through the pulmonary arteries may have contributed.

Bauser-Heaton et al. (14), through their meta-analysis, revealed lower mortality rates and a shorter duration of hospitalization for patients who underwent palliation using a PDA stent compared to those who received a systemic-pulmonary surgical shunt. However, it's important to note a higher incidence of reintervention in the PDA stent group. This increased need for reintervention in PDA stenting is unsurprising, given that these patients often undergo planned interval catheterization, leading to either scheduled or unplanned reinterventions during the procedure. The advantage of stent reintervention lies in its adaptability, functioning as an “adjustable shunt” that can be expanded as needed with the child's growth. Nevertheless, the decision for the preferred initial palliation strategy should be made cautiously, considering the potential implications of higher reintervention rates associated with PDA stenting, especially as the nature of these reinterventions—whether planned or due to clinically significant cyanosis—is often insufficiently reported in studies (14, 17–19).

One potential early reintervention scenario involves additional stent implantation due to incomplete ductal coverage by the initial stent. To mitigate this, prioritizing complete ductal coverage may be preferred, even if it results in stent protrusion into the pulmonary artery and the aorta. The most frequent reinterventions include stent redilation and new stent insertion to address reintervention, though there are instances where surgical revision or shunt placement becomes necessary if catheter-based reinterventions prove unsuccessful (14). Essentially, in the mBTT shunt procedure, any excess blood flow into the pulmonary artery, which could lead to overflow and trigger pulmonary reperfusion injury (saturation >85%), must be controlled through measures such as PDA ligation or MAPCAs unifocalization. Typically, mBTT shunts regulate pulmonary blood flow and oxygen saturation within the range of 70%–85%. However, in most cases, PDA ligation is omitted due to critically small pulmonary artery size or the use of a shunt graft smaller than required. Consequently, supplementary flow from alternative sources, such as the PDA, becomes necessary (20, 21).

### 4.2 Clinical outcome of ductal stenting vs. surgical shunting

Meadows et al. (22) found comparable mortality rates, transplant rates, and pulmonary artery growth between stent and shunt groups, albeit with a higher incidence of planned reinterventions in the stent group. Additionally, Prabhu et al. (23) demonstrated that PDA stenting was linked to reduced in-hospital morbidity and improved survival to stage II palliation in patients with single-ventricle physiology compared to surgical shunts. Thus, for this specific patient group, PDA stenting appears to be a comparable or potentially superior palliation strategy when compared to surgical shunting.

In our investigation, mortality rates were observed at 14% for PDA stents and 14% for m-BTT shunts. This figure exceeds the findings of Glatz et al. (5), where the mortality rate for m-BTT shunts was 10.4%, and for PDA stents, it was 6.6% (*p* = 0.26). The 30-day survival rate in our study was 86% for both treatment groups. This rate is lower than the survival rates reported by McMullan et al. (18), specifically 98% in the m-BTT shunt group and 92% in the PDA implantation group, although the differences were not statistically significant (*p* = 0.371) in neonates.

In cases where infants rely solely on the patent ductus arteriosus (PDA) for pulmonary blood flow (PBF), ductal spasm during the stenting procedure can swiftly escalate into a surgical emergency, leading to a complete halt in blood flow to the lungs (23–25). Consequently, some medical centers consider single-source PBF as a contraindication to PDA stenting, as reflected in the meta-analysis where surgical shunts were more commonly employed in such cases. Nevertheless, Bauser-Heaton et al. (14) discovered that among patients with single-source PBF, PDA stenting was associated with procedural complication rates and mortality comparable to those of surgical shunt procedures. This suggests that despite the rarity of ductal spasm, PDA stenting remains an appealing alternative for these patients. However, in this specific context, PDA stenting should be regarded as a procedure with relatively high risks, necessitating awareness from the cardiac intensive care team. Some centers may require more than one experienced operator and readiness to deploy surgical backup.

In a study conducted by Grozdanov et al. (26), a cohort of 130 patients was analyzed, comprising 49 who underwent PDA stenting and 81 who underwent mBTT shunt procedures. The study noted a higher incidence of acute procedure-related complications among patients who received ductal stenting compared to those who underwent mBTT shunt procedures (20.4% vs. 6.2%, *p* = 0.01). Additionally, 10 patients required subsequent shunt procedures following initial ductal stenting. The adoption of the less invasive PDA stent procedure and its associated shorter hospital stay were found to be counterbalanced by an increased requirement for stent reinterventions.

The ICU stay duration in the PDA stent group was shorter compared to the m-BTT shunt group [2 (0–16) vs. 4 (1–63), *p* = 0.002]. After multivariate analysis, it was determined that palliative measures influenced the length of ICU stay. Similar outcomes were also observed in the study by Glatz et al. (5), where the PDA stent group exhibited a shorter length of stay (5.3 vs. 9.1 days, *p* < 0.001). This finding aligns with expectations, considering the comparison involved both invasive and minimally invasive procedures.

### 4.3 Durability of ductal stenting vs. surgical shunting

Glatz et al. (5) demonstrated that there was no discernible difference between treatment strategies regarding their primary outcome hazard, which encompassed death or unplanned reintervention to address cyanosis. However, individuals treated with a PDA stent exhibited a lower risk of procedural complications, shorter length of stay in the Intensive Care Unit (ICU), and larger, more symmetrical pulmonary arteries before undergoing subsequent surgical repair or palliation.

This approach is, in part, influenced by the observation that patent ductus arteriosus (PDA) stents tend to undergo neointimal proliferation over time, potentially impacting their patency and durability, as emphasized by Sivakumar et al. (27) According to their report, patients with a two-ventricle anatomy initially treated with a PDA stent necessitate complete surgical repair earlier than the standard practice at their center due to in-stent stenosis and inadequate pulmonary blood flow. This tendency may be intrinsic to stent placement in ductal tissue, as there was no discernible difference in the use of antiplatelet or anticoagulation regimens between the groups. Despite these concerns, the duration from palliation to definitive surgical repair was notably longer in the PDA stent group, implying that a PDA stent can exhibit durability comparable to Blalock-Thomas-Taussig (BT) shunt placement, acknowledging the potential for elective PDA stent reinterventions in the interim. Although reinterventions were not identified as precursors to potential sudden cardiac death, they represent a tangible cost to the patient, both clinically and economically (5, 28, 29).

In this study, the reintervention rate stood at 2.3% in the PDA stent group and 7% in the m-BTT shunt group, though not significantly different (*p* = 0.436). Reintervention events were primarily attributed to thrombosis in both the PDA stent and m-BTT shunt groups. The reintervention rate for palliative measures in both PDA stents and m-BTT shunts was lower compared to the study by Lekchuensakul et al. (19), where it reached 8.8% in the PDA stent group and 15.6% in the m-BTTs group (*p* = 0.35). Factors contributing to elevated reintervention rates in that study included the presence of pulmonary artery branch stenosis, blood pressure variation measures, and body weight below 2.5 kg. In our study, the population's body weight exceeded 2.5 kg, and several patients underwent blood pressure variation procedures, resulting in a notably low reintervention rate.

## 5 Limitations

This study has inherent limitations due to its retrospective design and a relatively small cohort size. The cohort comprises high-risk individuals with diverse anatomical substrates. Furthermore, the lack of a standardized technique across all patients is attributed to the evolution of institutional practices over time.

## 6 Conclusions

In conclusion, this study offers valuable insights into the outcomes of late-presenting patients undergoing PDA stent procedures and m-BTT shunts, providing a comprehensive understanding of the clinical efficacy, complications, and cost considerations associated with these palliative strategies. The findings underscore the imperative for further research aimed at refining and individualizing the selection of palliative interventions tailored to the unique needs of this specific patient cohort.

Despite acknowledging the need for reinterventions in the PDA stent group, the study highlights the remarkable adaptability of stents and their potential benefits in accommodating the growth of pediatric patients. While recognizing the study's constraints, the results contribute essential knowledge to inform clinicians and enhance decision-making for this challenging and distinct patient population.

## Data Availability

The original contributions presented in the study are included in the article/Supplementary Material, further inquiries can be directed to the corresponding author.
